# Developing and Testing the *Populi Needle Exchange Point Finder*: An App to Reduce Harm Associated With Intravenous Drug Consumption Among Homeless and Non-homeless Drug Users

**DOI:** 10.3389/fpubh.2020.493321

**Published:** 2020-11-24

**Authors:** Fran Calvo, Xavier Carbonell, Carles Mundet

**Affiliations:** ^1^Departament de Pedagogia, Institut de Recerca sobre Qualitat de Vida, Universitat de Girona, Girona, Spain; ^2^Department of Quality Assessment, Evaluation and Research, Health and Community Foundation, Barcelona, Spain; ^3^Facultat de Psicologia, Ciències de l'Educació i de l'Esport Blanquerna, Universitat Ramon Llull, Barcelona, Spain; ^4^Servei de Promoció de la Salut a Girona, Public Health Agency of Catalonia, Girona, Spain

**Keywords:** drug addiction, harm reduction, eHealth, mHealth, mobile app, needle exchange programs, intravenous drug usage, homeless

## Abstract

The spread of viral infections remains a serious public health problem. People who inject drugs represent one of the highest-risk groups. eHealth and mHealth have been shown to be effective in improving individuals' management of their own health and their access to health care and to contribute to reducing the costs associated with certain medical interventions. People who inject drugs, including homeless people, tend to have access to technology. Young homeless people in particular are likely to use smartphones and social networking sites in ways that are similar to the general population. Despite this widespread use of technology, there are no apps designed specifically to reduce harm in people who inject drugs. The objective of this study is to analyze the development and usability testing process for an application for mobile devices, designed to complement the Needle Exchange Program. This app—the first of its kind—was developed by a public health agency, specialized professionals and people who inject drugs. We analyzed the differences in how health providers and drug users experienced the usability of the app. The participants were 61 members of multidisciplinary professional group and 16 people who inject drugs. We used a cross-sectional quantitative, observational design. First, we created and administered a questionnaire to collect the sociodemographic characteristics that could mediate the use of technology. Next participants tried the app and filled out a second questionnaire in which they rated their experience on a Likert scale from 1 to 7 in the following dimensions: overall attractiveness of the app, ease/difficulty of use, the extent to which they believed the app could improve access to injection materials, the extent to which they thought it would improve PWID's participation in the needle exchange program, overall utility, the degree to which they thought PWID would use the app, and the need for the app. To analyze the answers, we used contingency tables and compared means using a Student's *t* test. Finally, we conducted six audio-recorded focus groups about how the participants experienced the usability of the app. The objective of this part of the study was to classify and quantify the contributions of individuals and the group according to three predefined categories: potential benefits and positive aspects, potential obstacles or difficulties in carrying out the project, and concrete suggestions for improving the interface. There were not significant differences between the sociodemographic variables and the variables related to use of the app between professionals and PWID. Both professionals and PWID rated the app as intuitive and useful, especially the geolocation function for NEPs. Both groups also thought that the interface contained too much information and that this excess could be confusing for users. Both groups also had similar opinions about the app and its uses. An important difference between the two groups is that PWID reported that they would use the app, while professionals reported that they didn't think PWID would use it. All participants proposed improvements on the prototype, suggestions that will be applied in the creation of the definitive interface. Including professionals and patients in this sort of usability test enables researchers and developers to detect the needs of potential app users.

## Introduction

Intravenous drug consumption remains a serious public health issue. People who inject drugs (PIWD) are still among the social groups at greatest risk of contracting infectious diseases, including Human Immunodeficiency Virus (HIV) (1) and Hepatitis-C Virus (HCV) (2). In Spain and some other European countries, as many as 30% of PWID are infected with HIV. In Europe, Spain ranks second only to Germany in terms of HCV infection among this segment of the population, with a figure exceeding 80% (3). Additionally, Barcelona has the highest prevalence of fatal drug overdoses in Europe, and worldwide only a few US cities such as Chicago and Los Angeles have greater proportions of overdose deaths (4).

Needle Exchange Programs (NEPs) are highly effective in reducing the harm associated with drug consumption, especially in preventing the spread of infectious diseases and in reducing lethal overdoses (5). There is ample evidence that these programs are a cost-effective way to limit people's exposure to infectious diseases and to bring potential patients into contact with health services (6). NEPs improve patient adherence among a group that primary and specialized health services often face difficulties in retaining (7), and the programs tend to help curb the effects of intravenous drug use on the wider community (8).

Advances in information and communication technology (ICT) and the health-related applications of web 2.0 systems have meant that people are becoming ever more actively engaged in managing their own health (9). eHealth apps have proven to have potential as complementary tools in addiction treatment (10), even for patients at great risk of social exclusion, such as individuals experiencing homelessness (IEH) (11). While there is not yet enough scientific evidence to say conclusively which kinds of interventions are most likely to reduce addictive behavior, a number of eHealth studies have examined the use of mobile devices in the treatment of addiction and other health problems (10). IEH are among the groups who suffer the most from physical and mental health problems (12), and they are also especially likely to inject drugs (13). Recent studies have shown that both PWID (14) and IEH (15) are about as likely as the population at large to have mobile devices and Internet access. Notably, despite the overall similarities between the general population and the IEH population, the use of technology by IEH can be affected by some unique factors. For example, IEH that are housed at a shelter use technology more than people who live in public spaces, given that the latter live in situations of vulnerability that make it more difficult to acquire and maintain devices (16). At the same time, young IEH have greater access to owning and using smartphones than older IEH (17).

Despite this potential, we are aware of no apps that use technology to reduce the harm associated with the consumption of injected drugs or of app usability studies with professionals and PWID. Dependence on injected drugs means that PWID need sterile injection materials when they enter withdrawal and need to inject drugs. Lack of this material can lead the PWID to re-use his or her own injection material or, worse, share material with another user or rent it. In many countries, including Spain, injection material is free. The purpose of this article is to analyze the project that began with the creation of a smartphone app by a public health service. The main use of the app is to geolocate the position of the user with respect to the distribution and exchange points for injection material. Therefore, its application is the better distribution of this material and the reduction of harm associated with the consumption of injected drugs. The aim of this project is to forge a closer connection between PWID and NEP services in order to help bring about a reduction in the harm associated with intravenous drug use. To this end, we have: (i) developed an app for mobile devices aimed at both homeless and non-homeless PWID, (ii) tested the app's usability among health care providers and potential users, and (iii) assessed the feasibility of the app among providers and potential users.

## Materials and Methods

From January 2017 to May 2018, the Harm Reduction Team in the province of Girona (Catalonia, Spain) developed and tested the *Populi Needle Exchange Point Finder*, an app designed to provide PWID with better information about the NEPs near them. The chief function of the app was to determine the geolocation of the user with respect to the nearest NEPs and to provide users with information about these points. The software is a web-based app that can be accessed via any web browser or via a shortcut icon on the home screen of the user's mobile phone. The project was carried out in three phases. In Phase 1 we conducted a literature review of the published studies on similar apps and the development of an initial version of the app for testing purposes. In Phase 2, we carried out of a series of tests of the app's usability among health care service providers and potential homeless and non-homeless users. In Phase 3, changes were made to the app to implement the proposals made by the participants. The process of development and testing described in this article was generally based on the Unified Theory of Acceptance and Usability Technology model (18), which is organized around four constructs to explain the intention to use technology: performance expectancy, effort expectancy, social influence and facilitating conditions. The model uses mediating valuables such as gender, age, previous experience in technology use and the voluntary nature of use. Additionally, we used the guidelines for the development and usability of mobile apps for IEH set out by Sheoran et al. (19), who conducted the first usability test of a smartphone app among people at risk of extreme social exclusion.

### Procedure: Phase I. Literature Review and App Design

In February 2017, a comprehensive search was carried out in the PubMed and PsycINFO databases for scientific articles published in peer reviewed journals on the subjects of eHealth, harm reduction and intravenous drug use. The search terms included “harm reduction,” “needle exchange program,” “overdose,” “drug^*^,” and “homeless^*^,” in combination with the terms “mHealth,” eHealth,” “mobile phone,” “SMS,” and “Internet” (20).

Meanwhile, Author 1 conducted a series of interviews with experts in harm reduction, extreme social exclusion and ICT from the University of Barcelona, the Catalan Government's General Sub-Directorate on Drug Dependency and TIC-Salut (a company that provides the Catalan government with ICT services connected to the health sector) and two representative PWID users of NEP points in Catalonia contacted by outreach practitioners. Through informal, unstructured interviews we aimed to collect the experts' general impressions of the project and acquire specific contextual information that could complement what we discovered in our analysis of the literature.

Based on this initial work, we decided to develop a web-based app that would provide information on the locations of NEPs. We reasoned that such an app would not require users to occupy the memory of their mobiles, a potential obstacle for people with devices that were older or not fully functional. We also decided that the once the app had been downloaded it should work even if the device was not connected to the Internet, in order to ensure that users without data plans could access it. Finally, we determined that users should be able to choose between accessing the information anonymously and creating a profile. Regardless, all users would be able to post comments and write messages to other users and the app's administrators. For example, an anonymous user who had used the services of a specific NEP could provide feedback on his or her experience there by posting a comment on the NEP. The message would then be made visible to other users via the app's interface and to the administrators on an internal database. Messages and comments would remain visible for a limited period of time, to be determined by the administrator. The goal here is to encourage and monitor whether and how users contribute their opinions for the purposes of improving the app itself or enhancing their own experiences at NEPs.

**Figure 1:**
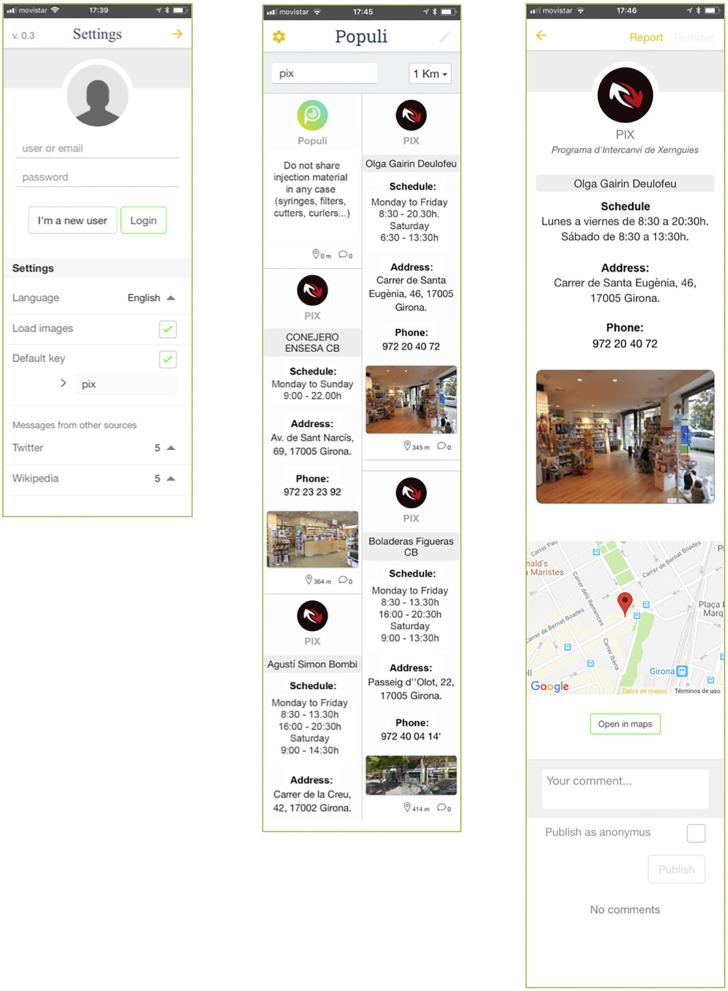
Screenshots of the testing version of Populi, before the modifications. Settings **(Left)**, Main interface **(Center**) and information about one of the NEPs **(Right)**.

The app was designed between the months of March and October 2017, and it features all the NEPs in the province of Girona, an area of Catalonia with over 700,000 inhabitants. The piloting of the app was conducted in the city of Girona, home to nearly 100,000 people. The NEP services in the city include primary care centers, treatment and harm reduction services, pharmacies and hospitals. The app provides opening hours, street views of the buildings, phone numbers and links to Google Maps to obtain directions. In addition, when PWID use the app, one of 25 different health tips is randomly displayed on the screen. Table 1 shows the technical aspects of the app. Figure 1 shows the initial design of the app, which participants tried in the usability test.

**Table 1:** Technical characteristics of the application.

**Populi App. Needle exchange program for intravenous drug users**
Localizer of registered Needle Exchange Points
Geolocation of the user with respect to the services located within a radius of kilometers selected by the user
Offline GPS function after initial download
Interactive map
Information about each center (hours, address, phone number and picture)
Access to a pop-up message featuring a randomly selected health tip each time the user opens the app (25 messages in total)
Link to the phone number for emergency medical services
Possibility of using the app as a registered or anonymous user
The possibility to post temporary comments for other users
User comments are saved in an Excel file by the developers
The user consents to the use of his or her data for research purposes upon accessing the app for the first time
Multilingual: Spanish, English and Catalan

### Instruments and Participants: Phase II. Usability Test

The usability test was administered in four settings between January and March 2018. The test was first given to professionals at a primary care center located in an area where intravenous drugs are often consumed on the street and where professionals regularly treat PWID (*n* = 13). The test was also administered at the Girona Health Promotion Service to 32 representatives of 27 different public entities involved in providing services for PWID and the homeless population. The test was also given to PWID in two settings, open outdoor areas where users consume intravenous drugs (*n* = 9) and an addiction treatment center (*n* = 7). The sampling was by convenience rather than probability. The inclusion criteria were having one's own smartphone and, in the case of users, being an active PWID.

For the purposes of recruiting professionals to take part in the study, a list was drafted of the agencies that deal with PWID and/or the homeless population, and one or more professionals from each of these entities was invited to participate. PWID were invited to take part in the study by the professionals at a public drug-treatment clinic and by the members of a harm reduction team. These participants were invited as they arrived at these centers, and they were offered economic compensation, which was provided to 16 individuals who took part in the study.

Before trying the app, participants filled out a sociodemographic questionnaire (gender and age for all participants, profession in the case of the professionals and condition of homelessness in the case of the PWID) and about their use of ICT tools, with an adapted version of Muscanell and Guadagno's test (21). Gender (22), age (23) and personal use of technology (24) are the chief variables linked to people's willingness to use eHealth tools. The conditions of the participants' homelessness were determined using the European Typology of Homelessness and Housing Exclusion, which classifies living situations as (i) rooflessness: without a shelter of any kind, sleeping rough, (ii) houselessness: with a temporary place to sleep in institutions or shelters, (iii) living in insecure housing: threatened with severe exclusion due to insecure tenancies, eviction, domestic violence, and (iv) living in inadequate housing: in caravans on illegal campsites, in unfit housing, in extreme overcrowding (25).

After the participants had completed the first questionnaire, they were asked to download the app and try it out. They were encouraged to consider how they would feel if using the app under the conditions faced by PWID, and they were asked to focus closely on all aspects of their user experience. All the participants received comprehensive instructions about the various sections of the app to ensure that they made full use of all its functions.

### Participant Written and Verbal Assessment of the App

After the test phase, the participants were asked to complete a second questionnaire with six items rated on a seven-point Likert scale related to (i) the app's overall appeal, (ii) ease/difficulty of use, (iii) the degree to which they believed the app could help provide easier access to materials needed for injection, (iv) the degree to which they believed it would improve the participation of PWID, (v) the overall degree of usefulness, (vi) how likely they thought the app's potential users would be to actually use it, and (vii) the degree to which they believed that an app of this sort was necessary. The professional participants completed two additional items: (vii) the degree to which they tend to ask their patients or the individuals they serve about ICT use, and (ix) the likelihood that their patients or the people they serve would use this app. Respondents were also asked, in the form of a yes/no question, whether they would recommend the app to their patients or the people they serve (professionals) or to friends and acquaintances (PWID).

Next, we invited participants to write individually their ideas about the app in the following three categories: (i) potential benefits and positive aspects, (ii) potential obstacles or difficulties in carrying out the project, and (iii) specific suggestions for improvements to the interface. Finally, we held four focus group sessions with professionals (with 13, 10, 11, and 11 participants, respectively; the first was specific to the primary care center) and two with PWID (of nine and seven participant, respectively), to talk about the application by discussing the three predefined categories about which they had written down their ideas.

### Data Analysis

In order to describe the results obtained, central tendency and dispersion measures were used for the descriptive variables, while absolute and relative frequencies were used for the categorical variables. Correlations were used to show the relationship between post-test variables and age. Depending on the normality of distribution, either Student's *t* test or U-Mann Whitney was used to show the relationship of these variables to gender and personal use of ICT.

The focus groups were led by authors 1 and 3 and two undergraduate research assistants. The sessions were audio-recorded. Authors 1 and 3 and an undergraduate research assistant listened to the audio material and grouped it into the three predefined categories. Because the groups were oriented toward analyzing specific aspects of the app, our objective was to analyze the concrete contributions to each category. We grouped responses under a set of codes within the three pre-defined categories. Once we had calculated the frequency for each of the codes in the three categories, we compared the results across professionals and patients. In the Results section, we have provided some of the written responses, as examples of the three categories.

## Results

### Description of Sociodemographic Data

The average age of the participants was 40.31 years (SD = 10.30). The participants from the group of PWID were younger than those from the group of professionals (*M* = 35.67 years old, SD = 5.61 vs. *M* = 41.83 years old, SD = 11.06, respectively, *t* = −2.18, df = 59, *p* = 0.033). The sample consisted of 21 men (34.43%) and 40 women (65.57), and differences associated with the respondents' gender were found among both professionals and PWID (*X*^2^ = 17.54, df = 1, *p* < 0.001).

Nineteen professionals (42.2%) worked at primary care centers and 15 (33.3%) worked at drug-treatment centers. Nurses made up 33.3% (*n* = 15) of the sample, while 24.4% (*n* = 11) were psychologists, 15.5% (*n* = 7) social workers and 13.3% (*n* = 6) doctors. All the professionals had smartphones. Among the PWID, 81.2% (*n* = 13) were experiencing homelessness and 14 (87.5%) had smartphones. The remaining participants (*n* = 2) were habitual smartphone users but did not have their own smartphone because they had lost or sold theirs in the preceding days. Taking into account the effort that these participants had made to attend the usability test, we decided to loan them a smartphone to carry out the test (Table 2).

**Table 2:** Characteristics of the sample.

**Characteristics**	**Professionals (*n* = 45)**	**PWID (*n* = 16)**	**Total (*n* = 61)**
	***n* (%) or *M* (SD)**	***n* (%) or *M* (SD)**	***n* (%) or *M* (SD)**
**Age**	41.83 (11.06)	35.67 (5.61)	40.31 (10.30)
**Gender**			
Man	8 (17.78)	13 (81.25)	21 (34.43)
Woman	37 (82.22)	3 (18.75)	40 (65.57)
**Workspace**			
Primary health care center	19 (42.22)	–	–
Drug treatment center	15 (33.33)	–	–
Homeless shelter	3 (6.67)	–	–
Pharmacy	3 (6.67)	–	–
University (academic)	2 (4.45)	–	–
Drop-in center	1 (2.22)	–	–
Long-term drug treatment center	1 (2.22)	–	–
Center for severe mental illnesses	1 (2.22)	–	–
**Profession**			
Nursing	15 (33.33)	–	–
Psychology	11 (24.44)	–	–
Social education/social work	7 (15.55)	–	–
Medicine	6 (13.34)	–	–
Pharmacy	3(6.67)	–	–
Others	3 (6.67)	–	–
Owned cellphone	45 (100)	15 (95.75)	60 (98.36)
Owned smartphone	45 (100)	14 (87.50)	59 (96.72)
Currently IEH or unstable housing	–	13 (81.25)	–

All the participants reported using apps and social networks, with 88.5% (*n* = 54) saying they did so on a daily basis. No differences were found across the two target groups in terms of the frequency of use of apps or social networks (Professionals = 86.7% and PWID = 93.7%), but there were differences in terms of the use of eHealth apps (Professionals = 44.4%, PWID = 6.2%) (Table 3).

**Table 3:** Personal use of ICT by group of participants.

**Variables**	**Total**	**Professionals**	**PWID**	***X*^**2**^**	**df**	***p***
	***n* = 61 (%)**	***n* = 45 (%)**	***n* = 16 (%)**			
**Use of apps**						
Daily	54 (88.5)	39 (86.7)	15 (93.7)	0.583	1	0.403
Weekly	7 (11.5)	6 (13.3)	1 (6.3)			
**Use of social network sites**						
Daily	54 (88.5)	39 (86.7)	15 (93.7)	0.583	1	0.403
Occasionally or never	7 (11.5)	6 (13.3)	1 (6.3)			
Use of eHealth apps (any degree of frequency)	21 (34.4)	20 (44.4)	1 (6.2)	7.63	1	0.004

In terms of the participants' opinions about the features of the app, reflected in their responses to the Likert-scale questions in the post-test, no differences were found between the target groups, nor were any found for gender, use of apps, use of social networks or use of eHealth applications (Table 4).

**Table 4:** Average post-test scores and participant characteristics.

		**Group**				**Gender**				**App personal use**				**SNS personal use**				**eHealth apps personal use**			
**Item**	**Total**	**Prof^**a**^**	**PWID^**b**^**	***t***	***p***	**Man**	**Woman**	***t***	***p***	**Daily**	**Weekly**	***z***	***p***	**Daily**	**Never^**c**^**	***z***	***p***	**Yes**	**No**	***t***	***p***
	***M* (DE)**	***M*** **(DE)**				***M*** **(DE)**				***M*** **(DE)**				***M*** **(DE)**				***M*** **(DE)**			
Degree of appeal	4.9 (1.4)	4.8 (1.3)	5.1 (1.6)	0.406	0.689	4.8 (1.4)	5.0 (1.4)	−0.516	0.609	4.9 (1.4)	5.2 (1.5)	−0.473	0.686	4.8 (1.3)	5.3 (1.6)	−0.900	0.387	4.8 (1.3)	5.0 (1.4)	−0.331	0.743
Degree of simplicity	5.7 (1.0)	5.7 (1.1)	5.8 (1.0)	0.255	0.80	5.7 (1.1)	5.7 (1.0)	0.026	0.979	5.7 (1.0)	5.5 (1.0)	−0.651	0.548	5.7 (1.1)	5.7 (0.49)	−0.236	0.828	5.9 (0.91)	5.6 (1.1)	1.16	0.250
Will improve access to injection materials	5.6 (0.99)	5.7 (0.9)	5.9 (1.1)	0.865	0.395	5.8 (1.0)	5.7 (0.99)	0.023	0.982	5.8 (1.0)	5.5 (0.55)	−0.950	0.383	5.8 (1.0)	5.7 (0.76)	−0.333	0.757	5.8 (1.0)	5.7 (1.0)	0.297	0.768
Users will post comments	5.5 (1.1)	5.4 (1.2)	5.7 (1.0)	1.09	0.282	5.6 (1.0)	5.5 (1.1)	0.470	0.641	5.5 (1.1)	5.3 (0.82)	−0.708	0.516	5.5 (1.1)	5.3 (1.1)	−0.811	0.454	5.5 (1.0)	5.5 (1.1)	0.213	0.833
Overall usefulness	5.7 (1.1)	5.6 (1.1)	6 (1.0)	1.27	0.211	5.7 (0.91)	5.7 (1.2)	0.206	0.837	5.7 (1.2)	5.7 (1.0)	−0.240	0.835	5.7 (1.2)	6.0 (0.58)	−0.524	0.638	5.7 (1.1)	5.7 (1.1)	0.188	0.852
Belief that it will be used	4.9 (1.1)	4.9 (1.1)	4.9 (0.95)	−0.187	0.853	5.1 (1.1)	4.8 (1.1)	0.941	0.353	4.9 (1.1)	4.8 (0.75)	−0.222	0.835	4.9 (1.1)	5.3 (0.49)	−1.15	0.282	4.9 (1.0)	4.9 (1.0)	0.182	0.856
Belief that the app was necessary	5.9 (0.95)	6.0 (0.92)	5.8 (1.0)	−0.631	0.534	5.8 (0.98)	6.0 (0.94)	−0.560	0.579	5.9 (0.99)	6.2 (0.41)	−0.382	0.741	5.9 (.99)	6.1 (0.69)	−0.396	0.722	5.9 (1.0)	6.0 (9.4)	0.005	0.996

a *Professionals*.

b *People who inject drugs*.

c *Occasionally or never*.

There was no correlation between the age of the participants and the mean scores they gave given to the app in terms of the overall appeal (*r* = 0.101, *p* = 0.447), the simplicity of use (*r* = 0.113, *p* = 0.394), the belief that the app would improve access to materials for injection (*r* = 0.055, *p* = 678), the belief that users would leave comments via the app about their experiences (*r* = 0.181, *p* = 0.171), the overall usefulness (*r* = 0.035, *p* = 0.790), the belief that the app is likely to be used (*r* = 0.014, *p* = 918) or the belief that it was necessary (*r* = 0.211, *p* = 0.109).

With regard to the questions that were asked only to the professionals, the average score for asking their patients about the use of ICT was 2.02 (SD = 1.37), while the score for the belief that patients would use the app was 3.02 (SD = 1.63). No differences were found among professionals related to their use of eHealth apps, to whether they asked about their patients' ICT use (Yes = 1.8, SD = 1.15 vs. No = 2.21, SD = 1.53, −0.982, df = 42, *p* = 0.332), or to the degree to which they believed that PWID would use the app (Yes = 2.85, SD = 1.39 vs. No = 3.17, SD = 1.83, −0.635, df = 42, *p* = 0.529). We found a correlation between the belief of the professionals that the patients would use the app in the future and the expressed interest of the PWID in using the app (*r* = 0.57, *p* < 0.001).

Finally, the participants contributed a total of 701 statements in response to the open-ended questions about the aspects of the app they found the most appealing and/or the app's potential benefits (*n* = 301, 42.9% of the statements), obstacles and difficulties (*n* = 180, 25.7%) and aspects in need of improvement (*n* = 220, 31.4%). The professionals contributed 495 of these statements, with an average of 11.0 per participant (SD = 4.2), while the PWID made 206 of the statements, with an average of 12.9 per participant (SD = 3.1).

### Insights From PWID

#### User Experience

*Populi* was deemed by these users to be simple and intuitive, and they were very appreciative that this tool allowed them to locate the nearest NEPs. The users gave especially high marks to the NEP map, rating this feature higher than the other information provided in the app. The PWID also appreciated that the project took into account the fact that they had mobile phones just like other members of society, highlighting that apps can be created for them just as they can for other segments of the population. As one of them said: “Finally, someone has realized that we have mobile phones too! The fact that we have this problem or that we live in the street doesn't mean that we aren't citizens or that we're not interested in our own health like anyone else.”

#### Feasibility

All the PWID who took part in the study believed that the app would be accepted by other PWID and IEH, and they all reported that they themselves planned to continue to use it after the study and that they would recommend it to other drug users: “I'm going to use [*Populi*] from now on. It will help me to find needles, because the place I usually go is always closed.” They reported that the web-app format might pose a problem if users were unfamiliar with how to save a shortcut to their home screen, and they recommended creating an informational campaign detailing the steps users would need to follow to download the app.

#### Suggested Changes

The most prominent concern was that the division of the app's main interface into two columns could confuse some users, and many participants said that there was too much information on each of the points. The address and phone number of each NEP were considered to be of less importance than the map, and they thought the map feature should be displayed more prominently. The PWID recommended standardizing the logo design for the various services and grouping them into categories (pharmacies, primary care centers and centers specifically devoted to harm reduction). They also suggested that it would be better if the main interface displayed only the NEPs that were open at the time of the search, thus filtering out unnecessary information: “I don't want to have to think too much when I'm searching for a needle. I want to open the app, see the nearest open point and what kind of service it is, then look at the map to find out how to get there. That's all I need from *Populi*.”

The users also suggested expanding the number of health tips and said that the app might also be useful as a platform for health providers to communicate with them and as a way to share information about topics such as vaccination campaigns, health official training, and overdoses.

### Insights From Professionals

#### User Experience

The professionals from the services also highlighted that the app was simple and intuitive. They were able to access all the app's features quickly and effectively: “*Populi* is very simple. In 5 min, you can familiarize yourself with all the options. It's very intuitive. You don't need a lot of instructions to be able to figure out how it works, pretty much from the very start.”

#### Feasibility

At the start of the study, 30% of the professionals did not believe that PWID had access to mobile phones. However, many thought that the GPS feature would be a suitable tool to improve access to the materials needed for injection. They also underlined that the app could have a potential benefit in improving the links between health services and PWID. They said that in addition to communicating information, the app might be used to post comments on users' experiences with NEPs, which they believed might help improve the relationship between these centers and the populations they serve. A total of 93.3% (*n* = 42) of the professionals stated that they would recommend the app to their patients: “From now on, I'll recommend [*Populi*] to all my patients who inject drugs. I think it's very important for them to have an app with up-to-date information about the NEP nearby. Additionally, the app lets them post their opinions about their experiences at the NEPs, which might provide us with some valuable information that could help us improve our harm reduction services.”

#### Suggested Changes

The professionals' suggestions for improvements to the app were along the same as those of the PWID. They emphasized that: (i) the two columns in the main interface might confuse some users, (ii) there was too much information about each NEP, (iii) the number of health tips could be increased, and (iv) it was important to give greater prominence to the map than to the rest of the information (Table 5).

**Table 5:** Contents of contributions made by participants and differences by target group.

	**Contents of proposals and contributions**	***n***	**Target group**		**Values**	
			**Prof.^**a**^**	**PWID^**b**^**	***X*^**2c**^**	***p***
			***n* (%)**	***n* (%)**		
Most appealing aspects. Potential benefits	Simple	49	36 (80.0)	13 (81.2)	0.085	0.539
	Listing of NEPs by distance	39	26 (57.8)	13 (81.2)	2.82	0.093
	Geolocation will improve access to injection materials	37	31 (68.9)	6 (37.5)	4.87	0.027
	Useful for improving links between PWID and NEP services	36	33 (73.3)	3 (18.7)	14.5	<0.001
	Intuitive	35	25 (55.6)	10 (62.5)	0.233	0.629
	The health tip is a good way to connect with PWID	35	29 (64.4)	6 (37.5)	3.50	0.061
	Access and opinions are anonymous	29	18 (40.0)	11 (68.7)	3.91	0.048
	Understands that PWID and homeless people have mobile phones like everyone else	20	9 (20.0)	11 (68.7)	12.7	<0.001
	Free	14	5 (11.1)	9 (56.2)	13.6	0.001
	Available in multiple languages	7	7 (15.6)	0 (0)	2.81	0.104
Main obstacles and difficulties	Too much information on the main interface	40	27 (60.0)	13 (81.2)	2.36	0.124
	The interface's distribution in two columns is confusing	38	27 (60.0)	11 (68.7)	0.385	0.535
	Some difficulties in downloading it as a web-app	29	18 (40.0)	11 (68.7)	3.91	0.048
	Access problems (phone signals, data plans)	17	3 (6.7)	14 (87.5)	38.4	<0.001
	PWIDs lack mobile devices	13	13 (28.9)	0 (0)	5.87	0.011
	PWIDs' lack interest. Reluctance to use the app	12	12 (26.7)	0 (0)	5.31	0.017
	Not useful if the PWID already regularly visits a specific NEP	12	12 (26.7)	0 (0)	5.31	0.017
	Irregular possession of mobile devices	11	5 (11.1)	6 (37.5)	5.56	0.028
	Professionals may not recommend or support the project	5	5 (11.1)	0 (0)	1.94	0.205
	PWID will not be open to receiving information via the app	3	3 (6.7)	0 (0)	1.12	0.394
Aspects to improve	Change the main interface so that there is only one column	50	35 (77.8)	15 (93.7)	2.04	0.146
	Give more prominence to the map than to the rest of the information about each NEP	36	21 (46.7)	15 (93.7)	10.8	0.001
	Use the same logo for each type of service, and identify the services more clearly	32	23 (51.1)	9 (56.2)	0.125	0.724
	Increase the number of health tips	23	19 (42.2)	4 (25.0)	1.49	0.222
	Increase the geolocation range beyond 30 km	19	14 (31.1)	5 (31.2)	0.072	0.595
	Highlight the NEPs that are open 24 h or nights and weekends	16	11 (24.4)	5 (31.2)	0.283	0.411
	Apply filters to order the information	15	12 (26.7)	3 (18.7)	0.399	0.395
	Reduce the amount of information about each NEP	9	0 (0)	9 (56.2)	29.7	<0.001
	Include the term “Pharmacy” before the name of each pharmacy	7	5 (11.1)	2 (12.5)	0.022	0.597
	Include information about the importance of returning used needles	6	6 (13.3)	0 (0)	2.37	0.147
	Show only the points that are open at the time of the search	4	0 (0)	4 (25.0)	12.0	0.001
	Make the user participation section more attractive	3	3 (6.7)	0 (0)	1.12	0.394

a *Professionals*,

b *People who inject drugs*,

c *Degrees of freedom = 1*.

### Phase III. Refinement

**Figure 2:**
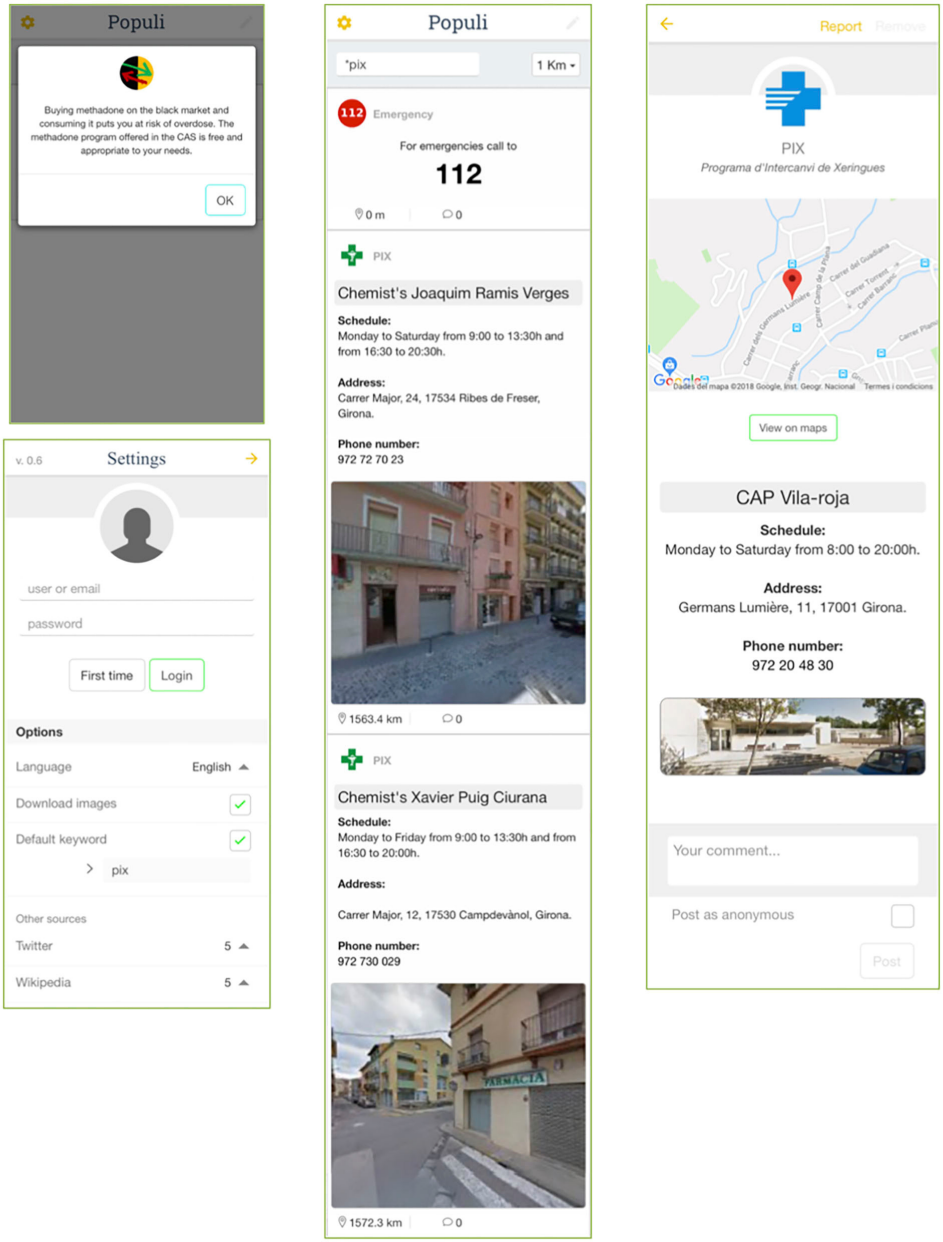
Screenshots of the interface after the implementation of the changes suggested during the usability test. Pop-up health tip **(upper left)**, settings **(lower left)**, main interface with geolocation results.

After an analysis of the proposed changes, modifications were made to the initial version of the app. Figure 1 shows the app's interface in its initial version, while Figure 2 displays the improved version incorporating the suggestions made during the user experience test. The modifications made included: (i) reducing the information displayed on the main interface (going from two columns to one), (ii) including the option to set the language to English, (iii) displaying the health tip as a pop-up message so that the user had to read it and close it before accessing the rest of the content, (iv) including the phone number for emergency services in a highly visible place at the top of the interface, (v) eliminating excess information and redesigning the presentation to lend greater prominence to the interactive map, over and above that of the written information, and (vi) creating a different icon for each kind of service.

## Discussion

We conducted usability testing of what we believe is the world's first NEP app for mobile devices, gathering data from people who inject drugs and the professionals who provide them services. Participants rated the app highly for user experience and feasibility. The results did not show a relationship between users' impressions of various aspects of the app and the potentially mediating variables of acceptance of eHealth, gender, age and personal use of apps. However, significant relationships were found between participants' responses to the app and their perceptions of the use of technology, and between professionals' stated interest in the ICT use of their patients and these professionals' tendency to think that PWID would use *Populi* [see also (24)]. The qualitative analysis of the potential benefits and suggestions for improvements to the app revealed similar responses across the two groups of participants. This feedback was used to optimize the app and ensure that the final version was an improvement over the version used for testing.

The aim of usability testing is to improve new applications by collecting the responses and experiences of potential users or of people involved in technical aspects of the apps. This type of test is believed to be an effective way to improve technology products aimed at specific target groups, and it is highly recommended that one be conducted before an app is launched (26). When dealing with highly vulnerable populations, especially those suffering from extreme social exclusion, such as homeless PWID, it is essential to work directly with the potential users of technologies in order to ensure the app's success (27). In fact, the PWID who took part in the study expressed appreciation for the fact that they were treated as active participants in the testing process. Mobile devices have been shown to play a role in the construction of identity (28), and nearly all of them highlighted that the project's developers had realized that they “had smartphones like everyone else.” This reflection flew in the face of the beliefs previously held by many professionals about the technology use of their patients who use intravenous drugs or are homeless. Professionals tended to be unaware that these patients use technology similarly to the rest of the population (15).

This study has a few limitations. First, the theoretical models that we have used will not be a perfect fit for all cases. However, used with care, such models can provide direction in studies of this kind. The Unified Theory of Acceptance and Usability of Technology encourages researchers to consider issues that might be overlooked without having a conceptual model. Furthermore, Sheoran et al. (19) offer a model of how to conduct research with difficult-to-reach populations, which can provide valuable information for developing public health proposals, in this case, focused on preventing the spread of viral infections. Second, the sample of users was smaller than the sample of professionals. This imbalance reflects the difficulty in finding active PWID who were able to participate, given their complicated symptoms and personal situations. Third, during the second test, the professionals were asked if their patients would use this app, reflecting professionals' perceptions rather than information about how users will actually behave or what they may need. In any event, the data reflect an interesting disparity between the perception of professionals and users about the future behavior of users. Fourth, the developers of *Populi* were the very professionals who work with intravenous drug users in the area where the project was carried out. This could have led to a social desirability bias among the participants. In order to neutralize this tendency to the extent possible, a neutral researcher administered the post-test in the cases in which this sort of bias was considered most likely, such as in the groups of PWID. Fifth, the test was conducted in a very small geographical area, which makes it difficult to determine how the app might work on a nationwide level. For this reason, research plans are now under way to test the app throughout the country. Finally, some of the potentially beneficial suggestions for improvements to the app were impossible to implement due to a lack of resources. For example, the PWID who participated in the test emphasized that only the NEPs that are open at the time of the search should appear on the display.

In conclusion, the PWID and service providers who participated in the test provided an overall vision of the app's usability, user experience and feasibility, allowing the developers to make significant improvements before offering it to the target population. In this way, we were able to detect errors and highlight areas in need of improvement and to produce an enhanced version of the app.

### Implications for Behavioral Health

Intravenous drug users remain at high risk of contagion for a number of viral infections, including HIV and HCV. NEPs have been shown to be effective in reducing the risk of contagion. Public health services have long struggled to communicate up-to-date information about the availability of harm reduction resources and NEPs to potential users. Now that eHealth and mHealth have become prevalent in many public and private health services and have shown a great deal of cost-effectiveness, this study shows that PWID and the professionals who serve them both request and appreciate the creation of health apps specifically for PWID. This study details the first-ever usability test of a geolocation app for NEPs. The involvement of PWID and professionals in the test and their specialized opinions as to how to improve the app's interface point the way forward for future interventions of this type to test eHealth and mHealth apps.

## Data Availability Statement

The datasets generated for this study are available on request to the corresponding author.

## Ethics Statement

The present study was approved by CEI-Girona Research Ethics Committee under the project code XSO_2017 on June 7, 2017 in accordance with the Declaration of Helsinki. The protocol of CEI-Girona included the standard of biosecurity and institutional safety procedures in concordance with the public health recommendations of the government of Catalonia. The participants were informed verbally and in writing of the objectives and methodology of the test, and they signed informed consent forms. The PWID received financial compensation in the amount of 15€ for their participation in the usability test.

## Author Contributions

FC performed testing, data collection, data analysis, and interpretation under the supervision of XC. FC, XC, and CM drafted the manuscript and provided critical revisions. All authors contributed to the study design and approved the final version of the manuscript for submission.

## Conflict of Interest

The authors declare that the research was conducted in the absence of any commercial or financial relationships that could be construed as a potential conflict of interest.
